# Current Trends in the Pharmacotherapy of Cataracts

**DOI:** 10.3390/ph13010015

**Published:** 2020-01-16

**Authors:** Segewkal H. Heruye, Leonce N. Maffofou Nkenyi, Neetu U. Singh, Dariush Yalzadeh, Kalu K. Ngele, Ya-Fatou Njie-Mbye, Sunny E. Ohia, Catherine A. Opere

**Affiliations:** 1 Department of Pharmacology & Neuroscience, School of Medicine, Creighton University, 2500 California Plaza, Omaha, NE 68178, USA; SegewkalHeruye@creighton.edu; 2 Department of Pharmacy Sciences, School of Pharmacy and Health Professions, Creighton University, 2500 California Plaza, Omaha, NE 68178, USA; MaffofouNgaelle@creighton.edu (L.N.M.N.); NeetuSingh@creighton.edu (N.U.S.); 3 College of Southern Nevada, Las Vegas, NV 89146, USA; Dariush.yalzadeh@csn.edu; 4 Department of Biology/Microbiology/Biotechnology, Federal University Ndufu Alike Ikwo, Abakaliki, Nigeria; Kalu.Ngele@tsu.edu; 5 Department of Pharmaceutical Sciences, College of Pharmacy and Health Sciences, Texas Southern University, Houston, TX 77004, USA; YaFatou.Njie-Mbye@tsu.edu (Y.-F.N.-M.); Sunny.Ohia@tsu.edu (S.E.O.)

**Keywords:** cataracts, lens, lens transparency, lens opacification, lens crystallins, antioxidants, glutathione, superoxide dismutase

## Abstract

Cataracts, one of the leading causes of preventable blindness worldwide, refers to lens degradation that is characterized by clouding, with consequent blurry vision. As life expectancies improve, the number of people affected with cataracts is predicted to increase worldwide, especially in low-income nations with limited access to surgery. Although cataract surgery is considered safe, it is associated with some complications such as retinal detachment, warranting a search for cheap, pharmacological alternatives to the management of this ocular disease. The lens is richly endowed with a complex system of non-enzymatic and enzymatic antioxidants which scavenge reactive oxygen species to preserve lens proteins. Depletion and/or failure in this primary antioxidant defense system contributes to the damage observed in lenticular molecules and their repair mechanisms, ultimately causing cataracts. Several attempts have been made to counteract experimentally induced cataract using in vitro, ex vivo, and in vivo techniques. The majority of the anti-cataract compounds tested, including plant extracts and naturally-occurring compounds, lies in their antioxidant and/or free radical scavenging and/or anti-inflammatory propensity. In addition to providing an overview of the pathophysiology of cataracts, this review focuses on the role of various categories of natural and synthetic compounds on experimentally-induced cataracts.

## 1. Introduction

Cataracts, one of the leading causes of preventable blindness worldwide, refers to lens degradation that is characterized by clouding, with consequent blurry or hazy vision [1]. There is evidence that cataracts account for 10.8 million out of 32.4 million blind individuals, and that 35.1 million out of 191 million people with impaired vision globally have this debilitating disease [2]. The prevalence of cataracts increases exponentially after 40 years of age, ranging from 3.9% among 55–64-year-olds to 92.6% among those 80 years and older [3,4,5]. In the US, the number of people suffering from cataracts is projected to double from 24.4 to 50 million by the year 2050 [6]. Epidemiological studies have elucidated common cataract risk factors to include age, smoking, ultraviolet (UV) radiation, female gender, steroid consumption, diabetes mellitus, and high body mass index [7,8]. As life expectancies improve throughout the globe, the number of people suffering from cataracts is predicted to increase worldwide [9], especially in low-income nations that lack easy access to cataract surgery, warranting a search for cheap, pharmacological alternatives to the management of this disease.

### 1.1. Classification, Signs and Symptoms of Cataracts

Cataracts are classified based upon the etiology of disease and anatomical location of opacity. Table 1 provides the etiological classification of various forms of cataracts as summarized by Gupta et al. (2014) [10]. Congenital and developmental cataracts occur during fetal growth or growth of children. Age-related cataracts are associated with old age and are mainly attributed to oxidative stress. Traumatic, complicated, and metabolic cataracts are attributed to physical trauma, inflammatory and degenerative eye disease, and metabolic disease, respectively. On the contrary, toxic, radiation and electrical cataracts occur due to exposure to toxicants, electromagnetic waves, and high electrical currents.

**Figure 1:**
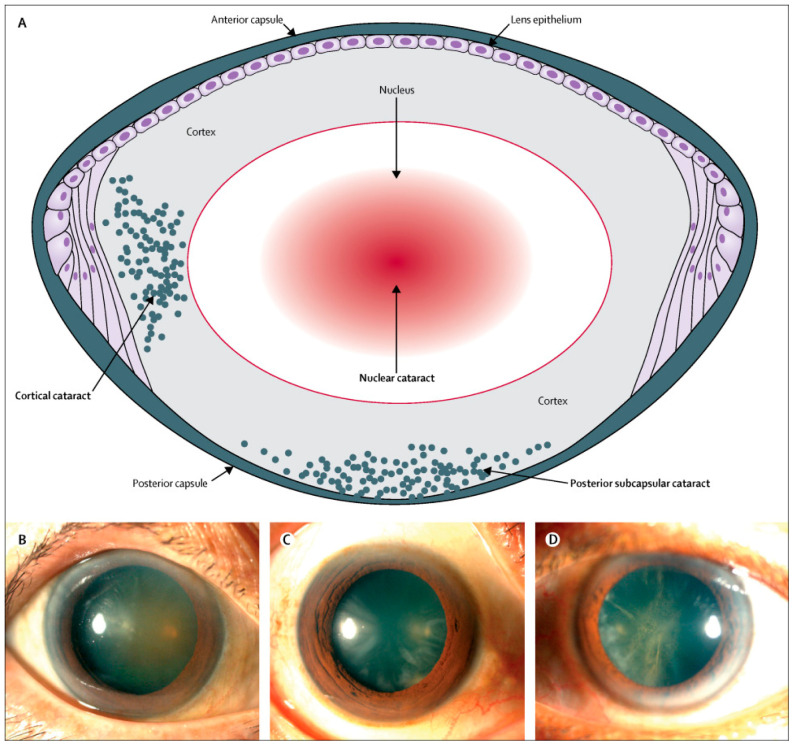
Characteristics of lens structures and major types of cataracts for location-based classification. (**A**) A schematic view of lens structures and corresponding types of cataracts. Slit lamp biomicroscopy photos showing (**B**) nuclear cataract, (**C**) cortical cataract, and (**D**) subcapsular posterior cataract. Copyright Elsevier (used with permission from Elsevier) [11].

**Figure 2:**
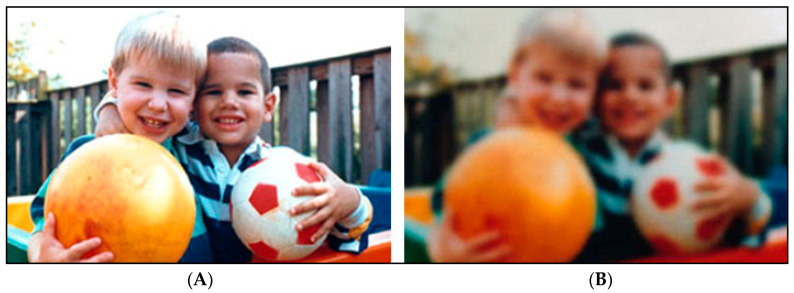
Scene viewed by normal vision (**A**) and a person with cataracts (**B**); sourced from NEI Media Library [13].

Age-related cataracts can be further classified based upon the anatomical location of opacity within the lens into nuclear, cortical, and posterior subcapsular cataracts (Figure 1A–D) [11]. Nuclear cataracts affect the center of the lens, with the lens becoming yellow or brown after hardening (nuclear sclerosis). Nuclear cataracts are common with older age and are associated with myopia. In contrast, cortical cataracts affect outer fibers of the lens (around edges of the nucleus) and assume a wedge-shaped appearance. Cortical cataracts can result in glares but do not alter vision as much as nuclear cataracts do. Posterior subcapsular cataracts, which affect the posterior cortex of the lens, are observed in relatively younger patients. This form of cataracts is associated with hyperopia and progresses faster than nuclear and cortical cataracts. Like cortical cataracts patients, patients with posterior subcapsular cataracts may experience glare. Posterior subcapsular cataracts are also associated with corticosteroid use [12]. Shared symptoms across different types of age-related cataracts include clouded or blurred vision (Figure 2), faded colors, glare, a halo around lights, poor night vision, and frequent prescription changes for corrective lenses [6,13].

### 1.2. Lens Anatomy and Physiology

**Figure 3:**
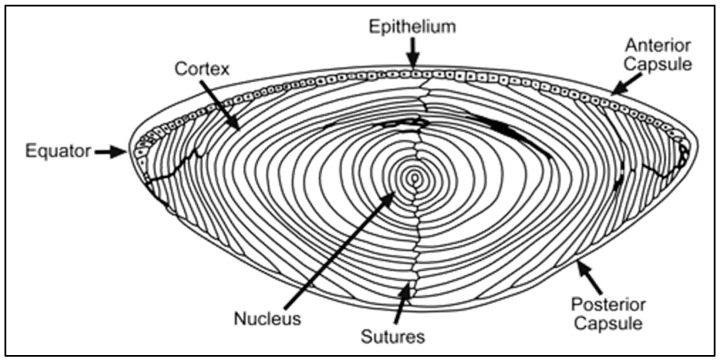
Schematic presentation of the cross-sectional view of mammalian lens. Used with permission from Roberts J [39].

A review of the anatomy and physiology of the lens is necessary to understand the pathophysiology of the disease. The lens is a transparent structure that is devoid of any blood supply. Anteriorly, the lens surface is covered by a monolayer of epithelial cells. In addition to maintaining lens metabolic activity, epithelial cells replicate to produce daughter cells, which migrate and differentiate into fiber cells [14]. Lens fiber cells make up greater than 95% of the lens and are stretched out to form compact, concentric layers (“shells”), thereby reducing intercellular space (Figure 3) [15]. Superficial lens fibers are nucleated and are metabolically active while deeper fibers, which make up most of the lens, are organelle-free with minimal metabolic activity. Interiorly, fiber cells have a high expression of soluble crystallin proteins but are devoid of nuclei, mitochondria, endoplasm reticulum, ribosomes, and other organelles [16]. Lens crystallins make up almost 90% of proteins in the mature lens [17]. In humans, the non-nucleated human lens fiber cells consist of α-crystallins, β-crystallins, and γ-crystallins (Table 2) [17]. Purification of high-molecular-weight α-crystallin fraction from human lenses yielded two homologous α-crystallin polypeptides: αA-crystallins and αB-crystallins [18,19]. The α-crystallin proteins account for up to one-third of the total protein in the lens [20]. However, three γ-crystallins (γC, γD, γS-crystallin), which are also found with five β-crystallin polypeptides (βB1, βB2, βB3, β A1/A3, βA4) predominate [21]. Table 2 provides a summary of crystallins identified in human lens according to size, amino acid residues, Gibbs free energy, their encoding genes, and chromosomal location [17].

The stability of soluble crystallins is regulated by several protective systems within and surrounding the lens. Acetylation of N-terminal residues in β- and γ-crystallins confer inherent protection of these lens proteins from exopeptidases. Additionally, acetylated N-terminal residues of α-crystallin subunits are buried in the interior of high-molecular-weight α-crystallin aggregate [22], rendering them inaccessible. The α-crystallin proteins prevent heat induced precipitation of β- and γ-crystallins [20] and serve as molecular chaperones that cloister misfolded proteins to mitigate pervasive protein aggregation [19]. Crystallin stability is further enhanced by the temperature difference in the anterior segment. Anterior portion of the eye is 2 °C lower than total body temperature due to evaporative cooling from cornea and distance from blood supply [23]. Additionally, ion pumps such as the Na^+^/K^+^ ATPase and Ca^2+^ATPase in pre-equatorial epithelium, together with gap junctions and channels such as aquaporin tightly regulate the homeostatic environment, thereby contributing the stability of lens crystallins [24]. Since deeper fibers which make up most of the lens are organelle-free, there is minimal metabolic activity and lower oxygen levels in the central part of the lens, thereby enhancing stability of lens proteins. Indeed, oxygen concentration in nucleus of lens has been reported to be as low as less than 10 µM in mammalian lens [25,26]. To this end, studies have shown that exposure of mammalian eyes to hyperbaric oxygen is associated with the development of nuclear opacities [27,28].

### 1.3. Lens Transparency

The lens is essential for focusing light onto the retina and can perform this function due to its transparent and dioptric properties. Transparency of the lens depends on avascularity, the paucity of organelles, narrow inter-fiber spaces, and regular organization of cells and proteins [29]. At the cellular level, there is a limited light-scattering by organelles due to their limited presence in the lens. Moreover, organelles are located away from the light path, exiled to the equator in the fibers from the central epithelium, thereby reducing light scattering in the lens. Transparency is also achieved by the short-range spatial order of proteins [30,31]. In fiber cells, crystallins are densely packed in a short-range order of about 250–400 mg/mL. The small protein size (<10 nm diameter), together with the close packing at high concentration, renders their wavelength less than that of light [17]. Furthermore, dense packing of protein aggregates reduces fluctuations of protein density and reduces the refractive index below wavelength of light [32]. Protein crystallization and precipitation are further deterred through a specialized mixture of crystallin protein forms (e.g., α, β, and γ forms), which confer superior solubility and native protein conformations in the lens [17]. In addition to their structural function within the lens, by increasing the refractive index, β- and γ-crystallins exhibit high solubility and thermodynamic stability to prevent scattering of light [22]. The α-crystallins serve as chaperones by partially binding to denatured proteins within the lens cells to form high-molecular-weight aggregates that maintain protein solubility and transparency [33].

In the cortex of the lens, transparency is enhanced by a high spatial order of fiber architecture with narrow intercellular spaces, which then compensates for light-scattering due to refractive index differences between membranes and cytoplasm. In the nucleus, high spatial order is not required due to minimal light scattering and negligible differences in the refractive index between fiber membranes and cytoplasm [15,34]. The cornea traps light with wavelength below 310 nm [35]. Interestingly, the mammalian lens possesses small-molecular-weight UV filters such as tryptophan metabolites (e.g., kynurenine, 3-hydroxykynurenine, 3-hydroxykynurenine glucoside) that remove UV radiation between 300–400 nm. Indeed, pathways for the biosynthesis of 3-hydroxykynurenine glucoside have also been identified in the mammalian lens [36,37,38].

## 2. Antioxidant Systems in the Lens

The lens is richly endowed with a complex antioxidant system consisting of non-enzymatic and enzymatic pathways, which further contribute to lens transparency. The major non-enzymatic antioxidants are glutathione (GSH), vitamin C (ascorbic acid), vitamin E, and carotenoids, while the enzymatic antioxidants include superoxide dismutase (SOD), glutathione peroxidase (GSH-Px), glutathione reductase (GSH-Rx), and catalase (CAT). Both non-enzymatic and enzymatic antioxidants scavenge reactive oxygen species to protect the lens. Failure in this primary antioxidant defense system leads to damage to lenticular molecules and their repair mechanisms. Ultimately, the degradation of lenticular molecules leads to cataracts [40,41,42,43].

### 2.1. Non-Enzymatic Antioxidants

GSH is a tripeptide thiol with a sulfhydryl residue that scavenges hydroperoxides and electrophilic compounds [44]. Lenticular GSH synthesis occurs in the cortex from the amino acids, glycine, cysteine, and glutamate. The enzyme, γ-glutamylcysteine synthase catalyzes the first and rate-limiting step in the biosynthesis of GSH and exerts feedback inhibition in response to GSH levels [44,45]. Incidentally, the lens has the highest concentration of GSH in the body [46], with the highest quantities being localized in the cortex, its site of synthesis [47]. GSH has a significant role in lens transparency by guarding membrane thiol groups which are vital in cation transport and regulation of the electrolyte balance [48]. Vitamin C is another non-enzymatic antioxidant that plays a vital role in the physiology of the lens. In a normal human lens, it is present at high concentrations of up to 3 mmol/L, a level which is 20–60-fold higher than that of the plasma content [49]. Similarly, α-tocopherol is also found in the normal human lens at 1.6 μg/g of lens tissue [49]. The only carotenoids found in the human lens are lutein and zeaxanthin. About 74% of these carotenoids are localized in the epithelium and cortex [50].

### 2.2. Enzymatic Antioxidants

Among enzymatic antioxidants, SOD removes superoxides through one electron dismutation of superoxide into H_2_O_2_ and oxygen. Three mammalian SOD isoforms have been described based on their structure, location, and metal-cofactors: the cytosolic copper-zinc (CuZn)-SOD (EC 1.15.1.1) or SOD-1, the mitochondrial (Mn)-SOD (EC 1.15.1.1) or SOD-2, and the extracellular (EC)-SOD (EC 1.15.1.1) or SOD-3, which also contains copper and zinc [51]. SOD-1 enzyme is the most prominent isotype in the lens [52]. The copper ion in the enzyme is involved in dismutation reactions undertaking alternate oxidation and reduction, while the zinc ion has only a stabilizing role with no activity in these reactions [53]. The H_2_O_2_ produced as a result of dismutation reaction is removed through the CAT and GSH-Px systems. GSH-Px removes H_2_O_2_ by using it to oxidize GSH, whereas CAT removes the H_2_O_2_ through the formation of oxygen and water [45,54,55]. Moreover, GSH disulfide (GSSG) is converted to a reduced form, GSH using GSH-Px enzyme and the co factor NADPH, from the pentose phosphate pathway [45,56]. Primate lenses have such robust protective antioxidant mechanisms that they can withstand incubation in H_2_O_2_ (1 mM), partly due to the high level of GSH-reductase (GSH-Rx) activity [57]. Additionally, there is a GSH-dependent thioltransferase system, including a GSH-S-transferase (GST) enzyme that repairs lens protein oxidation by cleaving protein–thiol groups to maintain a reduced state [58,59,60]. Lenticular thiol/disulfide homeostasis is upheld and the protein–protein disulfide bond is also maintained at low levels by the NADPH-dependent thioredoxin/thioredoxin reductase system [43,58,61,62]. Collectively, these protein and enzyme antioxidant systems play a vital role in further prevention of crystallin aggregation and the consequent development of cataracts [30,45,46].

## 3. Molecular Mechanisms of Cataract Formation

The molecular mechanism of cataract formation has been explained by the “protein aggregation” and/or “loss of protein solubility” disease models. The “protein aggregation” disease model hypothesizes that covalent bond damages lens crystallin proteins due to insult to systems that maintain redox, ionic, and other physiological environment leading to a partial unfolding of crystallin proteins. Research suggests that several mechanisms, including deamidation, oxidation, glycation, and truncation of crystallins, contribute to these covalent modifications associated with damage to lens crystallins [17,63,64,65,66,67,68,69,70,71,72,73,74]. Consequently, the crystallin proteins lose their native stability to become unstable molecules that aggregate, thereby shifting the refractive index. Thus, this model is based on loss of crystallin stability and the tendency of proteins to partially or fully unfold [17,75]. In support of this hypothesis, studies on materials from cataractous lens have revealed the presence of multiple species of lens proteins with higher molecular weight forms due to aggregation and polymerization. These high molecular weight aggregates cause light scattering in the lens, thereby accounting for lens opacity [63,64,65,66,67,68,69,70,71,72,73,74]. The “protein aggregation” cataract disease model is contrary to that proposed for other protein aggregation diseases such as Alzheimer’s disease, Huntington’s disease and Parkinson’s disease, where the high molecular weight proteins further aggregate to form plaque deposits in the affected parts of the CNS [17].

The “loss of solubility” model hypothesizes that aberrant interactions of native crystallins lead to loss of solubility of crystallins in their native conformations, with consequent precipitation into protein-rich and protein-poor regions. This ultimately disrupts the short-range order to alter the refractive index of the lens [75,76]. Some studies have proposed that altered solubility and crystallization propensity account for hereditary cataract in certain disease states [76,77,78,79].

## 4. Treatments Strategies for Cataracts

### 4.1. Current Cataract Treatments

Up to date, there is no pharmacological intervention available for the management of cataracts, leaving surgery as the main option for the treatment of this disease. Among the different surgical approaches, phacoemulsification is a technique that involves the use of a probe that emits ultrasound to break up the lens, which is then aspirated through a thin cannula introduced with a smaller incision in the cornea. Extracapsular cataract extraction, which involves the removal of nucleus and cortical materials, leaving behind the posterior capsule, is commonly used in developed countries. This procedure is used for advanced cataract that cannot be removed with phacoemulsification [80]. While intracapsular surgery is not routinely used, it involves surgical removal of the entire lens with its capsule [81]. Even though cataractous lenses can be surgically removed and replaced with an artificial intraocular lens, a procedure that has been successful in restoring sight, there are still barriers to cataracts surgery and eye care in many countries. Indeed, access to surgery remains a challenge in developing nations, where cataracts are estimated to accounts for 90% of total blindness among those of lower socioeconomic status [9]. Additionally, intra-operative (e.g., posterior capsular rapture, zonular weakness, suprachoroidal hemorrhage) and post-operative complications (e.g., posterior capsular opacification, elevated IOP, persistent anterior uveitis, retinal detachment) still occur [82]. Thus, pharmacological strategies to management of cataracts may play a significant role in regions of the world where surgical options are limited.

### 4.2. Potential Pharmacological Treatments for Cataracts

Based on the models of cataract development and the proposed mechanism for its formation, there have been attempts to use herbal and/or antioxidants, amino acids, and minerals to attenuate the development of cataracts. Interference with protein aggregation pathway and modulation of chaperone activity to promote refolding are also among the efforts that have been attempted. Furthermore, patented approaches involving the following mechanisms are available: inhibition of glycation-specific mechanisms, phase separation inhibitors, modulators of the TGF-β pathway, and inhibition of matrix metalloproteinase [83]. This section provides a review of different categories of compounds that have been evaluated for anti-cataract potential, in vitro, ex vivo and in vivo.

#### 4.2.1. Antioxidants

The role of oxidative stress in the onset and progression of cataractogenesis has been well described in literature [40,70]. Consequently, antioxidants and free radical scavengers present a potential therapeutic strategy to the management of cataracts.

(i) In vitro and ex vivo studies

Typical antioxidants

There is evidence that depletion of GSH affects lens transparency and leads to the development of cataracts. Indeed, lenticular GSH is essential in preventing oxidative stress elicited by H_2_O_2_, where it serves to maintain lens protein sulfhydryl groups in a reduced state, thereby averting formation of inter- and intra-molecular disulfide cross-linkages. GSH also plays a protective role in membrane permeability and active transport of lens protein sulfhydryl groups. It regulates calcium influx and protects lenticular protein from oxidative and osmotic stress [84,85,86]. Indeed, GSH mitigated lipid peroxidation and enhanced the activity of SOD, CAT, and GSH-Px enzymes in H_2_O_2_ (10 mM)-induced cataract formation in cultured goat lenses, ex vivo [87]. There is evidence that endogenous antioxidants, vitamin C, and GSH attenuated structural crosslinking, oligomerization, and proteolytic instability of lens crystallins in a lens protein solution exposed to sunlight in the presence of a photosensitizer, riboflavin [88]. Taken together, these studies affirm the protective role of GSH in the pathophysiology of the lens.

The carotenoids, lutein and zeaxanthin, and the vitamin E derivative, α-tocopherol, mitigated an H_2_O_2_-induced increase in protein oxidation, lipid peroxidation, and DNA damage in human lens epithelial cells. Moreover, these antioxidants also increased GSH and the GSH:GSSG ratio [89,90]. Interestingly, the vitamins C and E but not the carotenoids, lutein and zeaxanthin, conferred resistance in H_2_O_2_-induced depletion of GSH in epithelial cells, in vitro, suggesting that protective effect of carotenoids was not dependent on the elevation of GSH [89,90]. The vitamin E derivative, α-tocopherol, prevented the formation and progression of glucose-induced cataract in goat lenses incubated in artificial aqueous humor. This activity was further supported by the higher Na^+^-K^+^-ATPase activity and low lipid peroxidation compared to glucose-only treated lenses [91].

Amifostine, an organic thiophosphate prodrug, is clinically used as a cytoprotective adjuvant in cancer chemotherapy due to its free radical scavenging capacity and ability to detoxify reactive drug metabolites [92]. Belkacémi et al. (2001) reported that the active metabolite of amifostin, aminothiol WR-1065, protected bovine epithelial cells against X-ray radiation of 10 Gy (2 Gy per minute) as evidenced by the elevation of GSH content, enhanced cell viability, and a decrease in both nuclear condensation and epithelial cell apoptosis [93].

In the past few decades, an endogenously-derived organosulfur compound that serves as an essential co-factor for several enzymes in the body, α-lipoic acid, has emerged as a potent antioxidant, nutritional supplement, and an aldose reductase inhibitor [94,95]. There is evidence that α-lipoic acid elicits its antioxidant activity by serving as a free radical scavenger and/or stimulating the biosynthesis of endogenous antioxidants such as GSH [96,97,98]. To this end, α-lipoic acid mitigated H_2_O_2_ (0.2 mM)-induced lens opacification, apoptosis and lipid peroxidation in cultured Sprague–Dawley rat lenses, ex vivo. Moreover, in biochemical studies, it enhanced the activity of the endogenous antioxidants, SOD and GSH-peroxidase in peroxide-treated lens homogenates [99].

Keto acids and amino acid antioxidants

There is evidence that the ketoacids, pyruvate and α-ketoglutarate, which are intricately involved in several metabolic pathways in the body, possess antioxidant activity in various biological systems [100,101,102]. Oxaloacetate functions as an intermediate in several metabolic pathways, including gluconeogenesis, the glyoxylate cycle, amino acid synthesis and fatty acid synthesis [103]. Both pyruvate and oxaloacetate serve as precursors for the synthesis of several essential and non-essential amino acids. Interestingly, pyruvate (10 mM), α-ketoglutarate(20 mM), and oxaloacetate (20 mM) decreased the level of lenticular lipid peroxidation and enhanced activity of GSH-peroxidase in H_2_O_2_ (10 mM)-induced cataract formation in cultured goat lenses [104]. Similarly, pyruvate attenuated in H_2_O_2_ (10 mM)-induced lipid peroxidation and enhanced SOD, CAT, and GSH-peroxidase activity in cultured goat lenses [87]. Moreover, pyruvate prevented H_2_O_2_-induced insolubilization of ɣ-crystallin content in rat organ culture system [105], thereby affirming the protective role of ketoacids in mammalian lens physiology.

In addition to the ketoacids, amino acids and their derivatives have also been reported to exert a protective effect on mammalian lenses. Amino acids make a vital contribution to lens clarity because they serve as precursors for the biosynthesis of lens proteins (such as GSH, crystallins). Indeed, insufficient ingestion of certain amino acids has been linked to cataractogenesis in rats [106]. Rathore and Gupta (2010) evaluated the effect several amino acids, glycine, l-tryptophan, l-tyrosine, l-phenylalanine, l-histidine, l-lysine, l-arginine, l-cysteine, l-glutamic acid, l-aspartic acid, and l-proline on H_2_O_2_ (1 mM)-induced cataract formation in goat lenses for 24 h. With the exception of l-tyrosine and l-phenylalanine, all amino acids tested conferred significant protection from peroxide-induced GSH depletion. Moreover, all amino acids except l-aspartic acid elevated total soluble protein content in peroxide-treated lenses. Taken together, these data support a protective function for amino acids in the mammalian lens, ex vivo [107].

l-cysteine is an essential amino acid that serves as a precursor for the biosynthesis of the tripeptide thiol antioxidant, GSH. *N*-acetylcysteine, a prodrug for l-cysteine, is well established as a thiol antioxidant with better bioavailability compared to the parent amino acid. *N*-acetyl-cysteine is clinically versatile, being used as an antidote for acetaminophen poisoning and a mucolytic agent, amongst others [108]. In experimental animals, *N*-acetylcysteine has been shown to replete hyperoxia-induced reduction of GSH in rat liver and mitigate hyperglycemia-mediated protein oxidation in cultured rabbit lens cells, in vitro [109,110]. *N*-acetylcysteine amide, a derivative of *N*-acetylcysteine, exhibits similar antioxidant activity but superior accessibility to intracellular tissues due to its lipophilicity [111]. Wang et al. (2009) investigated the effect of *N*-acetylcysteine amide on hyperoxia-induced cortical opacification in adult New Zealand rabbit lenses, ex vivo. These workers reported that *N*-acetylcysteine amide (20 and 40 mM) prevented hyperoxia-induced cortical opacification and reduction in Na^+^-K^+^-ATPase activity. Additionally, this compound elicited an elevation in soluble proteins, GSH and GSH-Rx activity [112]. In other studies, *N*-acetylcysteine amide protected rat lenses from dexamethasone-induced cataract formation with a corresponding elevation in the GSH:GSSG ratio, GSH-Rx activity, and a reduction in lipid peroxidation [113], thereby affirming the protective role of *N*-acetylcysteine amide on cataract formation, ex vivo.

Acetyl-l-carnitine is an amine precursor for carnitine, a key player in fatty acid metabolism within the cell. There is evidence that acetyl-l-carnitine confers protection from oxidative stress in several biological systems [114,115]. Acetyl-l-carnitine mitigated selenite-induced cataractogenesis in Wistar rat lenses, ex vivo. Moreover, the activities of CAT, GSH-Px were higher and lipid peroxidation lower in acetyl-l-carnitine treated lenses, affirming its protective function in selenite-induced cataractogenesis [116]. Propolis, a compound produced by bees, reduced reactive oxygen species and increased cell viability in rat epithelial cell cultured with glucose, implicating the role of propolis in inhibiting hyperglycemia insult-initiated oxidative and osmotic stress [117].

Plant-derived compounds and herbal extracts

There is a large body of evidence supporting the anti-cataract potential of numerous medicinal plants, presumably via mechanisms that involve antioxidant potency of these plant-derived compounds and/or extracts. Quercetin, a flavonoid that is present in many fruits, vegetables, and grains, has been identified as a potent antioxidant and anticancer agent [118,119]. Quercetin prevented cataract progression and formation in glucose-induced goat lenses incubated in artificial aqueous humor in a manner that was characterized by a decrease in lipid peroxidation and an increase in Na^+^-K^+^-ATPase activity [91]. Chrysin, a flavone found in honey, prevented selenite-induced cataract formation in cultured Wister rat lenses by modulating genes responsible for calcium transport, calpain activation, and apoptosis [120]. Chaudhury et al. (2017) also showed that flavonoids from green tea prevented UV radiation-induced oxidative damage to human γB-crystallin, in vitro [121]. Moreover, a polyphenol derived from green tea, epigallocatechin-3-gallate (EGCG) attenuated and reversed the aggregation of αA(66–80), a peptide fragment derived from αA-crystallin peptide. Since αA(66–80) aggregates play a role in the destabilization of native crystallin structures, EGCG can potentially prevent onset and reverse cataract formation [122]. In corroboration, green tea *(Camellia sinensis)* leaf extract mitigated Na selenite-induced reduction on SOD, GSH-Px and CAT activities, and increase in lipid peroxidation in Wistar rat lenses, ex vivo [123]. In general, these studies suggest a protective role for natural polyphenols and flavonoids in cataract formation.

Drevogenin D is a triterpenoid aglycone derived from *Dregea volubilis*, a plant which belongs to the *Asclepiedaceae* family. Extracts from *Dregea volubilis* have found medicinal application for various conditions such as inflammation, asthma, dyspepsia, tumors [124,125,126]. Biju et al. (2007) demonstrated an anti-cataract activity of the triterpenoid aglycone, drevogenin D from *Dregea volubilis*, in a selenite-induced Sprague–Dawley rat cataract model, ex vivo. Not only did the presence of drevogenin D mitigate selenite-induced inhibition of SOD, CAT, and GSH-Px enzyme activities, it also elicited an increase in GSH and sulfhydryl levels and a decrease in lipid peroxidation [127].

Fenugreek (*Trigonella foenum-graecum*), a leguminous plant that is used globally as a spice, has been shown to possess multiple beneficial medicinal properties such as antidiabetic, anticarcinogenic, hypocholesterolemic, antioxidant, and immunological effects [128]. Moreover, the extracts of fenugreek seed have been shown to possess potent antioxidant and free radical scavenging activities in vitro and in vivo [129,130]. Using Wistar rat lenses, Gupta et al. (2010a) demonstrated that the aqueous extract of fenugreek (*Trigonella foenum-graecum* seeds) could sustain lens transparency and restore lenticular activities of SOD, GSH-Px, CAT, and GST enzymes in a selenite-induced lens model. Moreover, this plant extract also blocked selenite-induced depletion of GSH and higher lipid peroxidation in Wistar rat lenses, ex vivo [131]. Using the same experimental model, Gupta and co-workers also found that the aqueous extract of the herbal preparation Triphala, made from three fruits of Asian origin, *Emblica officinalis, Terminalia chebula*, and *Terminalia belerica* protected cultured rat lenses from selenite-insult by maintaining optical clarity, restoring the activity of SOD, GSH-Px, CAT, and GST and attenuating lipid peroxidation [132]. Although the exact composition of Triphala is variable, this herbal mixture has been reported to exert multiple beneficial effects in humans [133].

*Moringa oleifera*, a plant belonging to the *Moringaceae* family, has found therapeutic application in a wide range of conditions including epilepsy, fever, hypertension, inflammation, tumors, hyperlipidemia, and diabetes. Due to enrichment of vitamins A and C and minerals, the *Moringa oleifera* plant cans serve as a nutritional supplement and a source of natural antioxidants in malnourished individuals [134,135]. The ethanol extract of *Moringa oliefera* prevented lens opacification of hyperglycemia (55 mM)-induced cataract formation up to 72 h. Moreover, *Moringa oleifera* extracts restored GSH, soluble protein content, CAT activity, and mitigated lipid peroxidation, ex vivo [136].

*Abutilon indicum*, a plant that belongs to the *Malvaceae* family, is an indigenous shrub found in tropical and subtropical climates in Asia. It is used as a herbal remedy for a wide range of ailments such as fever, infection, and inflammation [137,138,139]. Hydroethanolic extracts of *Abutilon indicum* effectively attenuated lens opacification in glucose (55 mM)-induced cataract formation in cultured goat lenses. In these studies, the extract-treated lenses displayed a reduction in malondialdehyde content and an increase in total protein content and SOD activity. Moreover, the extracts attenuated aldose reductase activity in hyperglycemia-treated lenses, thereby affirming its anti-cataract activity in an acute diabetic cataract lens model, ex vivo [140]. In other studies, the ethanol extract of ginger *(Zingiber officinale)*, a well-known spice, delayed hyperglycemia-induced opacification in cultured goat lenses, ex vivo. The ginger extract treated lenses also displayed a higher Na^+^-K^+^-ATPase activity and total protein content, and a reduction in lipid peroxidation [141].

*Hippophae rhamnoides* L. is a deciduous shrub of the family *Elaeagnaceae* that is also known as Seabuckthorn. Although exact composition varies with geographical location of the plant, the seabuckthorn plant is enriched with minerals, vitamins, and micronutrients with medicinal and nutritional value [142]. Leaf extracts of seabucthorn have been shown to possess potent antioxidant activity in C-6 glioma cells, in vitro [143]. The aqueous extract of seabuckthorn leaf delayed initiation and progression of H_2_O_2_-induced cataract formation and restored GSH, SOD activity in goat lenses, ex vivo [144].

*Abies pindrow*, commonly known as west Himalayan fir, is an evergreen tree that belongs to the *Pinaceae* family. Leaf extracts of *Abies pindrow* with demonstrated potent antioxidant activity [145], have been used as a herbal remedy for various ailments, including fever, diabetes, bronchitis, and antispasmodic [146]. Dubey et al. (2015a) investigated the anti-cataract activity of the aqueous leaf extract of *Abies pindrow* on H_2_O_2_-induced cataract formation in goat lenses, ex vivo. These investigators reported that the herbal extract prevented clouding caused by peroxide insult and enhanced GSH and total protein contents and SOD activity, when compared to peroxide-only treated lenses [147]. In another study, Dubey et al. (2015b) further delineated the role of *Luffa cylindrica* on cataract formation in goat lenses, ex vivo. Also known as sponge gourd, *Luffa cylindrica* is a fibrous plant belonging to the family *Cucurbitaceae*. In addition to being consumed globally as a vegetable, the fruit possesses potent antioxidant properties and is used as a traditional medication for inflammation and diabetes management, amongst others [148,149,150]. Dubey (2015b) and coworkers showed that a standardized preparation of *Luffa cylindrica* fruit extract protected goat lenses from H_2_O_2_-induced opacification, lipid peroxidation, and enhanced total protein content, ex vivo [151].

*Ocimum tenuiflorum* (also known as *Ocimum sanctum*) is a perennial plant that is indigenous to Indian subcontinent that belongs to the *Lamiaceae* family. *Ocimum sanctum* is used as a herbal remedy for various ailments, including inflammation (e.g., arthritis), respiratory problems (e.g., cough, asthma, bronchitis), malaria, stomach problems (e.g., diarrhea), and ocular pain in some Asian nations [152]. Gupta et al. (2005) found that aqueous extracts of *Ocimum sanctum* could mitigate selenite-induced damage to proteins and antioxidant enzymes in rat lenses, ex vivo [153].

In summary, a wide range of compounds and herbal mixtures that possess antioxidant properties have been shown to protect lenses from oxidative damage, ex vivo and in vitro. For these compounds to be clinically significant, these studies need to be replicated in vivo. Furthermore, extensive chemical characterization of the active compounds in the plant-derived products needs to be performed to determine their pharmacological mechanism/s of action. Table 3 provides a summary of antioxidant drugs that elicit an anti-cataract effect on various models of the disease, in vitro and ex vivo.

(ii) In vivo studies

Various experimental animal models, including the inducible or genetic models of cataracts have been employed in studies of cataractogenesis, in vivo [154,155,156]. The induced cataract types include models for oxidant stress-induced (selenite, naphthalene, I-buthionine-(S,R)-sulfoximine (BSO), hyperbaric oxygen), diabetic cataract (streptozotocin, galactose, and rat strains with spontaneous onset diabetes); UV-induced cataract (exposure to UVA and UVB) and steroid-induced opacification in different animal species [154,155,156]. Using these in vivo models, different antioxidants have been evaluated for their anti-cataract activity.

Typical antioxidants

Lenticular vitamin C is a crucial antioxidant and ultraviolet-filter that mitigates entry of light rays into the lens, thereby minimizing consequences of oxidative damage to the lens [157,158]. Insufficient consumption of vitamin C elicited a reduction in lenticular levels of ascorbic acid in guinea pigs [159], while dietary intake of the vitamin was found to increase lenticular ascorbic acid levels in rats [160,161]. Peighmbarzadeh and Tavana (2014) investigated the effect of vitamin C on selenite-induced cataractogenesis in New Zealand albino rabbits, in vivo. In this study, cataract was induced by a single subcutaneous injection of selenite (20 μmol/kg) on the 10th postpartum day. Daily subcutaneous injection of vitamin C (2.5 mg/rabbit) from the eighth day (postpartum) for 14 days attenuated cataract formation in 40% of animals [162]. In support of these observations, biochemical studies revealed a higher level of total soluble protein content compared to selenite-treated animals, thereby affirming its anti-cataract activity [162]. Using a similar experimental model, Jahadi Hosseini et al. (2008) demonstrated a 50% protective action for vitamin C (0.3 mM) from selenite-induced cataract formation in Sprague–Dawley rat pups, in vivo. Biochemical assays confirmed a higher concentration of total proteins and soluble proteins in vitamin C-treated animal lenses, compared to that of selenite-only treated animals [163]. Moreover, vitamin C attenuated galactose-induced cataract formation in guinea pigs [164]. Recently, this vitamin was reported to mitigate selenite-induced cataract formation and the corresponding loss in chaperone activity in Sprague–Dawley rat pups, in vivo [165]. Taken together, these studies suggest a partial protective role of vitamin C in experimental cataractogenesis, thereby affirming the multifactorial etiology of the disease. On the contrary, higher levels of vitamin C have been shown to serve a pro-oxidant role in mammalian lens, presumably due to its oxidative stress metabolites such as dehydroascorbic acid [166].

The fat-soluble vitamin, vitamin E, has also been reported to serve as a crucial antioxidant in the lens defense system. Not only is a deficiency of vitamin E linked to an increase in the risk of cataractogensis in rats [167,168], but its anti-cataract activity has been demonstrated against selenite-, galactose-, steroid-, streptozocin- and UV radiation-induced cataract in experimental animals [165,167,168,169,170,171]. Amongst possible mechanisms, vitamin E has been shown to elevate GSH levels and protect the chaperone activity of crystallin proteins, thereby preserving the antioxidative defense mechanism within the lens [165,169].

Khan et al. 2017 showed that the potent, endogenous disulfide antioxidant, α-lipoic acid, prevented the onset and progression of fructose-induced cataract in Sprague–Dawley albino rats. These workers also found an increase in the level of several antioxidants (GSH-Px, CAT, SOD, and GSH) while lipid peroxidation was reduced, compared to that of fructose-only treated animals. Interestingly, α-lipoic acid treatment reinstated lens protein Ca^2+^ ATPase and Ca^2+^ in the lens [172]. In other studies, the anti-cataract effect of α-lipoic acid together with pantethine, penicillamine, and deferoxamine were attributed to their antioxidant effects [173,174,175,176].

The potent pyridoindole antioxidant and free radical scavenger, stobadine, deferred the progression of streptozocin-induced diabetic cataract in Wistar rats by blocking lipid peroxidation, in vivo [171,177]. In other studies, the endogenous free radical scavenger, melatonin, attenuated BSO-induced cataract formation in neonatal rats in vivo and enhanced total lenticular GSH content, suggesting that either the free radical scavenging and/or a stimulatory effect on GSH content was responsible for the anti-cataract activity [178]. Taken together, these studies affirm the protective role of antioxidants on the pathophysiology of the lens, in vivo.

The dietary minerals, zinc and copper, have been reported to prevent cataract formation. Indeed, copper and zinc are crucial for the activity of the lenticular antioxidant systems such as the cytosolic SOD-1 enzyme [179,180]. Low lenticular concentrations of zinc have been observed in patients with mature age-related cataracts and high levels in those with traumatic cataracts [181]. In a recent study, dietary supplementation with zinc for a period of six weeks mitigated the formation of streptozocin-induced cataracts in experimental rats [182]. In addition to enhanced levels of α-crystallin proteins, zinc supplementation attenuated the lenticular expression of polyol pathway enzymes [182]. Topical application of zinc sulfate eye drops has also been reported to attenuate lens opacity induced by intravitreal injection of sodium selenite in adult rabbits, in vivo [183], supporting an important role for zinc in lens clarity.

Although selenium is a trace element that plays a significant role in lenses where it supports the activity of antioxidant selenoproteins such as GSH-Px [184], toxic levels of this element produce oxidant stress-mediated cataract formation, leading to its application in the induction of experimental cataracts [154,156]. Karakucuk et al. observed a decrease in the concentration of selenium in the aqueous humor and lens of patients with age-related cataracts, an important finding that supports the significant role of selenium in the activity of GSH-Px enzyme in maintaining lens clarity [185]. Ebselen, an organoselenium molecule that possesses antioxidant, neuroprotective, and anti-inflammatory activities, protected Sprague–Dawley rat pups from selenite-induced cataracts, in vivo [186,187]. Moreover, the ebselen-treated lenses exhibited clearer lenses, higher lenticular GSH content, and less lipid peroxidation when compared to that of selenite-only treated animals [187]. Taken together, these studies confirm the vital antioxidant role of trace elements in maintaining lens transparency, in vivo.

Ketoacids and amino acids antioxidants

The ketoacid, pyruvate, attenuated the progression of streptozotocin- and selenite-induced cataracts in experimental animal models, in vivo [105,188]. In addition to exhibiting anti-cataract activity, ex vivo [112], *N*-acetylcysteine, a prodrug for the amino acid and thiol antioxidant l-cysteine, has been shown to replicate its anti-cataract activity in vivo. *N*-acetyl-cysteine inhibited selenite-induced nuclear opacities in Sprague–Dawley rats, in vivo [189]. Lenticular malondialdehyde and protein carbonyl levels were lower in lens homogenates from *N*-acetylcysteine-treated rats while GSH content was significantly higher compared to that of control animals [189]. Tuzcu et al. (2014) sought to determine the role of intravitreal triamcinolone acetonide injection on cataract formation in albino Wistar rats, in vivo. Although there was no visible lens opacification in response to steroid exposure, *N*-acetylcysteine protected lenses from the steroid-induced decline in GSH and GSH-Px activity and a corresponding decline in malondialdehyde and protein carbonyl content, compared to those of control animals [190].

*N*-acetylcysteine amide is a more lipophilic derivative of *N*-acetylcysteine, with superior bioavailability into intracellular sites. Both *N*-acetylcysteine amide and glutathione ethyl ester eye drops delayed onset of streptozotocin-induced cataract formation in Sprague–Dawley rats, as evidenced by the high level of thiol and catalase activity in lens homogenates. Interestingly, both drugs were unable to delay progression or reverse lens opacity in the advanced stages of the disease [191], presumably due to the depletion of the total lenticular antioxidant pool. Maddirala et al. (2017) also investigated the effect of both topically- and intraperitoneally-administered *N*-acetylcysteine amide on selenite-induced cataract formation in male Wistar rat pups [192]. In this experimental model, Na selenite (19 μM/kg body weight) was administered intraperitoneally on 10th postpartum day, intraperitoneal *N*-acetylcysteine amide (250 mg/kg body weight) on ninth, 11th and 13th postpartum days, while topical *N*-acetylcysteine drops (1%) was instilled into the eyes from postpartum 15th and 30th days. Interestingly, both intraperitoneal and topical *N*-acetylcysteine amide attenuated the severity of cataracts formation, in vivo. Furthermore, the amide-treated lens homogenates exhibited higher levels of GSH content, thioltransferase and m-calpain activity, while lipid peroxidation, GSH-Rx activity, and calcium levels were reduced when compared to selenite-only treated lens homogenates [192]. Carey et al. (2011) also investigated the effect of *N*-acetylcysteine amide on cataracts induced by the GSH synthesis inhibitor, BSO, in a Wister rat pup model of the disease. Slit lamp examination of animal lenses revealed that *N*-acetyl-cysteine amide prevented cataract formation in 80% of lenses. Biochemical studies revealed a reduction in lenticular protein carbonylation and lipid peroxidation while antioxidant defense enzymes were replenished, compared to that of BSO-only treated lenses [193]. In agreement with data from ex vivo studies [116], intraperitoneal administration of acetyl-l-carnitine prevented selenite-induced cataractogenesis in Wister rat pups, via mechanisms involving inhibition of lipid peroxidation and restoration of antioxidant content and activity, in vivo [194]. *N*-acetylcarnosine, a prodrug of l-carnosine also attenuated onset and progression of age-related cataracts in humans and dogs [195], corroborating its effect in lenticular epithelial cells, propolis, delayed onset and progression of galactose-induced cataract Sprague–Dawley rats, in vivo [117].

Plant-derived compounds and herbal remedies

Plant-derived compounds and herbal remedies have also been reported to demonstrated anti-cataract potential in studies using experimental animals with this disease. The fruit and vegetable-derived flavonoid, quercetin, is a potent antioxidant and free radical scavenger that possess several health benefits, including cardioprotective, anti-diabetic, anti-inflammatory, and anti-cancer effects, amongst others [196]. As observed in ex vivo studies [91], quercetin prevented the onset and progression of selenite-induced cataracts and sustained lens chaperone activity in Sprague–Dawley rats, in vivo [165]. In other studies, intraperitoneal injection of a citrus flavonoid, rutin blocked selenite-induced lenticular opacification in Wistar rats, with a corresponding elevation in the activity of the antioxidant enzymes, CAT, SOD, GSH-Px, GST, and GSH-Rx and a reduction in lipid peroxidation, compared to selenite-only treated lenses [197].

Hesperetin is a flavonoid found in orange rinds that has been reported to elicit beneficial cardiovascular and anti-inflammatory action effects [198,199]. Hesperetin prevented selenite-induced cataract formation in rats by attenuating the loss of the lens protein, filensin, and replenishing GSH and ascorbic acid levels [200]. Furthermore, hesperetin and its derivatives, hesperetin stearic acid ester and hesperetin oleic acid ester, counteracted the decrease in α-crystallin chaperone activity due to selenite, in vivo [201,202].

Ellagic acid, a polyphenol found in several plants and fruits such as grapes, nuts, strawberries can elicit antiproliferative and antioxidant effects, in vitro and in vivo [203,204,205]. Sakthivel et al. (2008) showed that ellagic acid mitigated selenite-induced opacification in Wistar rats, in vivo. Ellagic acid treated lenses exhibited superior CAT, GSH-Px, SOD, GST activities, compared to selenite-only treated control lenses. Moreover, malondialdehyde and calcium level was lower in ellagic acid treated lenses compared to untreated selenite group [206].

The leaves and leaf buds of the evergreen shrub, *Camellia sinens* of the family *Theaceae*, have been used for centuries to produce the beverage known as tea. Both green tea and black tea, which are rich in polyphenols, prevented the onset and progression of lens opacification by restoring antioxidants in selenite-induced and streptozotocin-induced cataract [121,123,207,208]. The biological effects of caffeine, a methylxanthine alkaloid found in plants and used as a beverage worldwide, has been extensively described in literature [209,210]. There is evidence supporting a protective role for caffeine from UV radiation-induced cataractogenesis, in vivo. Indeed, topically administered caffeine mitigated UV radiation-induced cataractogenesis and lens apoptosis in Sprague–Dawley rats [211,212,213]. Moreover, coffee delayed onset of selenite-induced cataract in rats and preserved antioxidant enzymes and lens protein [165,214]. Caffeine and pyrocatechol also sustained lens chaperone activity in selenite-induced Sprague–Dawley rat lenses, in vivo [165].

Other dietary antioxidants have been extensively explored in the prevention of cataract formation, in vivo. The carotenoids (α-carotene, β-carotene, lutein, lycopene, and cryptoxanthin) were shown to elicit a protective action from both oxidative stress- and hyperglycemia-induced cataract formation, in vivo [165,215,216,217]. Nakazawa et al. (2017) also demonstrated that both oil-soluble (e.g., β-carotene, lutein, zeaxanthin, anthocynain) and water-soluble (e.g., cyanidin) antioxidants could impede the onset and progression of selenite-induced cataracts and sustain lens chaperone activity in Sprague–Dawley rats, in vivo [165]. Curcumin is a bright yellow compound with antioxidant properties and is produced from the *Curcuma longa* plant belonging to the *Zingiberaceae* family. Curcumin delayed the initiation and progression of galactose-, oxidative stress- and streptozotocin-induced cataract formation, in vivo, by mechanisms that involved the preservation of lenticular antioxidant, lipid peroxidation, and soluble protein content [218,219,220,221].

Resveratrol (3,5,4′-trihydroxy-trans-stilbene) is a natural phenolic stilbenoid present in the skin of grapes, blueberries, raspberries, mulberries, and peanuts [222,223]. Resveratrol has been reported to possess health benefits in several chronic diseases such as diabetes mellitus, various cancers, metabolic syndrome, cardiovascular diseases, and Alzheimer’s disease, to mention a few [223,224]. Resveratrol prevented selenite-induced cataract in Sprague–Dawley rat pups, as evidenced by the optical clarity of lenses and higher GSH and reduction in lipid peroxidation in resveratrol-treated lenses [225].

*Heliotropium indicum*, also known as Indian heliotrope, is a plant that belongs to the family *Boraginaceae.* In some parts of Asia, its leaf extracts are used as a traditional remedy for wounds, skin ulcers, conjunctivitis and cataracts, amongst others [226,227]. Whole plant aqueous extracts of *Heliotropium indicum* prevented selenite-induced cataractogenesis in Sprague–Dawley rats, in vivo. In addition to preserving aquaporin 0, crystallins (αA &αB), total lens proteins, and GSH contents, its aqueous extracts attenuated lipid peroxidation, suggesting an antioxidant basis for its anti-cataract activity [228].

*Echium amoenum*, a perennial plant that also belongs to the *Boraginaceae* family, has been shown to possess antimicrobial, antidiabetic, and anxiolytic properties, amongst others [229]. Hydroalcoholic extract of *Echium amoenum* also elicited a protective effect against selenite-induced lens opacification in rats, in vivo. These investigators concluded that the antioxidant phytochemicals within the plant accounted for the anti-cataract activity of *Echium amoenum* [230].

IH636 grape seed proanthocyanidin extract (GSPE), an oligomeric proanthocyanidin extracted from grape seeds, has been shown to possess potent free radical scavenging and antioxidant properties with several potential health benefits [231,232]. IH636 GSPE attenuated cataract formation in Sprague–Dawley rat pups, most likely through the antioxidant actions of the extract [233].

*Cassia tora*, also known as *sennatora*, is a herbaceous plant that belongs to the *Fabaceae* family, has a wide application ranging from edible vegetable, food additive, medicinal (laxative, treatment for skin disease, antiparasitic) to industrial (cassia gum—a thickener and gelling agent) use [234]. *Cassia tora* leaves delayed the onset and progression of cataract formation in neonatal rats. In addition to preserving the antioxidant defense system, lens morphology and the expression of lens crystallins, *Cassia tora* attenuated lipid peroxidation, compared to selenite-only treated lenses.

*Vaccinium corymbosum* (northern highbush blueberry), a deciduous shrub that belongs to the family *Ericaceae*, has been shown to be rich in vitamin C and polyphenol antioxidants [235]. A decoction of *Vaccinium corymbosum* leaf attenuated the onset and progression of selenite-induced cataracts in neonatal rats [236]. Lenses from the decoction-treated animals exhibited significantly higher levels of antioxidant markers and soluble proteins while protein aggregation and activation of calpain were mitigated [236].

*Emilia sonchifolina* (also known as “lilac tassel flower”), a tropical plant that belongs to the *Asteraceae* family, is used as a herbal remedy for ocular ailments, fever, sore throat, diarrhea, eczema, and as an antidote for snake bites in some Asian countries [237,238]. Lija et al. (2006) found that *Emilia sonchifolia*-derived flavonoids prevented the initiation and progression of selenite-cataract formation, in vivo. In addition to enhancing activities of SOD, CAT, GSH enzymes, the flavonoids reduced lipid peroxidation compared to selenite-only treated animals, suggesting that antioxidant mechanisms account for the anti-cataract effect of *Emilia sonchifolia*-derived flavonoids.

Broccoli (*Brassica oleracea var. italic*), a common vegetable that belongs to the family *Brassicaceaeis,* is naturally enriched with nutritional antioxidants and flavonoids [239]. Broccoli has been reported to possess several health benefits such as decreasing the risk of cancer [240]. Broccoli-derived flavonoid fraction attenuated the onset and progression of selenite-induced lens opacification with corresponding higher GSH content, activity of antioxidant enzymes (CAT, SOD) and a reduction in lipid peroxidation, calcium, and calpains when compared to selenite-only treated animals [241].

Lupeol, a pentacyclic triterpenoid present in the flavonoid fraction of *Vernonia cinereal* plant that belongs to the *Asteraceae* family, possesses antimicrobial, anti-inflammatory, and anticancer activities [242]. Asha et al. (2016) demonstrated an anti-cataract action for lupeol in selenite-induced Sprague–Dawley rat pups, in vivo. Compared to selenite-only treated animals, lupeol attenuated selenite-induced lipid peroxidation as well as the decline in the activity of the antioxidant enzymes, CAT, GSH-Px, GSH-Rx, GST, and Ca^2+^ATPase [243].

*Allium sativum* (garlic) and *Allium cepa* (onion) are bulbous plants that belong to the *Amaryllidaceae* family. Commonly used as a seasoning worldwide, the therapeutic potential of garlic has been reported to include anti-tumor, cardioprotective, anti-diabetic, antimicrobial, amongst others [244]. In addition to delaying onset of streptozotocin-induced cataract in Wistar rats, the methanolic *Allium sativum* (garlic) extract mitigated hypoglycemia-induced oxidative stress associated with the disease in a dose-dependent manner and restored lenticular SOD and GSH-Px activities and GSH content [245]. In other studies, the flavonoid-rich, aqueous extract of *Allium cepa* (onion), preserved optical clarity in selenite-treated Wistar Albino rats, in vivo. Moreover, the extract treated lenses exhibited higher total antioxidant level, GSH content, and higher activity of GSH-Px and SOD enzymes compared to untreated lenses, in vivo [246].

*Pinus densiflora*, a pine tree that belongs to the family *Pinaceae*, is naturally enriched with antioxidant procyanidins and polyphenols [247]. *Pinus densiflora* has been used as a traditional remedy for inflammation and pain in some Asian countries [248]. The bark extract of *Pinus densiflora* prevented selenite-induced cataract development in Sprague–Dawley rat pups in a dose-dependent manner [249], with a corresponding enhancement of lenticular water-soluble protein and GSH contents; antioxidant activities of SOD, GSH-Px, and CAT enzymes; and a reduction in water-insoluble protein content and lipid peroxidation. Furthermore, the extract counteracted the selenite-induced downregulation of αA-crystalline, lens-specific m-calpain (Lp84), filensin and phakinin, and the antiapoptotic factor (Bcl-2), and the upregulation of apoptotic proteins [249]. In other studies, *Ocimum sanctum* delayed the onset and incidence of selenite-induced cataracts, and preserved antioxidants and soluble protein contents in rat lenses, in vivo [153].

*Crocus sativus*, commonly known as saffron crocus, is a flowering plant that belongs to the *Iridaceae* family. The *Crocus sativus* stigma, which is enriched with nutrients such as flavonoids, crocetin, and crocin is widely used as a spice and a traditional medicinal remedy for various ailments such as stomach pain [250]. Aqueous extract of *Crocus sativus* stigma prevented selenite-induced cataracts in rats and enhanced the antioxidant enzymes SOD, CAT, GSH-Px, and GSH content. Additionally, the extract attenuated lenticular selenite-induced lipid peroxidation, protein oxidation, and proteolysis, thereby affirming the antioxidant-based mechanism for the anti-cataract action of the plant [251].

The plants, *Pterocarpus marsupium* and *Trigonellafoenum-graceum*, which belong to the *Fabaceae* family are found to have traditional application as anti-diabetic remedies [252]. Therefore, Vat et al. (2004) sought to investigate the possible application of these plants to prevent diabetic cataracts and found that the aqueous extracts of the bark of *Pterocarpus marsupium* and the alcoholic extract of the *Trigonellafoenum-graceum* seeds displayed an anti-cataract effect in alloxan-diabetic rats, in vivo. Moreover, the anti-cataract potency was directly proportional to the hypoglycemic potency of the plants, leading the authors to conclude that the hypoglycemic capacity of the plants was responsible for the anti-cataract effect observed [253].

*Ginkgo biloba*, a tree from the family *Ginkgoaceae*, has been used for centuries as a traditional remedy for various ailments such as cough and asthma in some Asian countries [254,255]. Oral supplementation with an extract of *Ginkgo biloba* delayed the onset and progression of cranial radiation-induced cataract formation and abolished the corresponding reduction in SOD and GSH-Px activities and increase in lipid peroxidation in Sprague–Dawley rats, in vivo [256]. For further details, the reader is referred to the rigorous review by Thiagarajan and Manikandan, focused on the anti-cataract activity of vitamin C, vitamin E, and curcumin along with prototype plant materials with antioxidant activities tested against cataracts [257].

Gupta et al. (2010) investigated the pharmacological actions of Triphala, consisting of *Emblica officinalis, Terminalia chebula*, and *Terminalia belerica* on selenite-induced cataract in Wistar rats in vivo. In corroboration with its ex vivo effects, the herbal combination blocked the progression and intensity of selenite-induced cataracts, in vivo [132]. In summary, various plant extracts and products that possess antioxidant properties have tremendous potential in the development of anti-cataract medications. Table 4 provides a summary of antioxidant drugs that elicit an anti-cataract effect on various models of the disease, in vivo.

#### 4.2.2. Non-Steroidal Anti-Inflammatory Drugs (NSAIDs)

In the 80′s NSAIDs emerged as potential anti-cataract agents after aspirin was found to have anti-cataract effect in a rheumatoid arthritis study [258]. Subsequently, aspirin and variety of NSAIDs such as ibuprofen, naproxen, sulindac, bendazac, 5-hydroxybendazac, and bendazac-lysine were pursued for their anti-cataract activity [259,260,261,262,263,264,265,266,267,268]. These observed anti-cataract activities of NSAIDS were due to different mechanisms such as acetylation, inhibition of glycosylation, and carbamylation of lens proteins [259]. Sulindac possessed anti-cataract activity, possibly due to aldose reductase inhibition [269] while naproxen had effect on galactose-induced and selenite-induced cataract in rats [270,271]. Naproxen reversed depletion of GSH and high malondialdehyde levels due to oxidant stress induced lipid peroxidation in isolated rat lens [272]. Although some studies implicate the inhibition of aldose reductase for the anti-cataract activity of NSAIDs other studies indicate antioxidant properties, suggesting an array of pathways [7,259,260,261,262,263,264,265,266,267,268,269,270,271]. However, no recent studies have surfaced in the past few years in supporting an anti-cataract role for NSAIDs besides their common post-cataract surgery use to prevent cystoid macular edema [273,274].

#### 4.2.3. Miscellaneous Drugs

In addition to antioxidants, various miscellaneous compounds have exhibited an anti-cataract effect. Pharmacological chaperons that bind α-crystallins (cryAA and cryAB) reversed protein aggregation in various models of cataracts. For example, compound 29 (5-cholesten-3b,25-diol) prevented protein aggregation and enhanced protein solubility, thereby improving lens clarity in severe age-related cataracts (R120G cryAB knock-in mice), hereditary cataract due to a mutation (R49C cryAA knock-in mice), and age-related cataract (C57BL/6J wild-type mice) [275].

Angiotensin-converting enzyme (ACE) inhibitors, lisinopril and enalapril, have been reported to elicit anti-cataract activities in glucose-induced cataract, probably due to the antioxidant and free radical scavenging mechanisms [276]. Enalapril and lisinopril were also able to retard the glucose (55 mM)-induced opacification in goat lenses cultured in artificial aqueous humor, ex vivo. Contrary to ACE-treated lenses in which Na^+^-K^+^-ATPase activity and total protein content was higher, the glucose-only treated lenses displayed a higher level of lipid peroxidation [276].

Ursodeoxycholic acid, a component of bear bile, similarly prevented selenite-induced cataract formation on M-199 cultured Wister rat lenses, ex vivo [277]. Hydrogen saline and ursodeoxycholic acid were able to elevate the total antioxidative capabilities and activity of SOD, CAT, GSH-Px, GSH-Rx, and GST compared to untreated lenses following selenite insult, ex vivo. Moreover, the contents of sulfhydryl groups and GSH were higher in hydrogen saline- and ursodeoxycholic acid-treated lenses as compared to the selenite-only treated group, which displayed higher lenticular malondialdehyde [277,278].

Posterior capsular opacification is a common secondary complication to cataract surgery that involves intraocular lens implantation with an artificial intraocular lens [279]. Sternberg et al. (2010) investigated a method to prevent secondary cataract in New Zealand rabbits after implantation of intraocular lens, in vivo [280]. Compared to control animals that developed opacification in six weeks, these investigators demonstrated that treatment with methotrexate and actinomycin D mixture loaded hyaluronic acid delayed opacification up to six months, postoperatively [280]. In other studies, a sustained cyclosporin A delivery microsphere prevented postoperative posterior subcapsular cataract development in New Zealand white rabbits, in vivo [281]. Brown and Akaichi (2015) observed an association between vitamin D deficiency and posterior subcapsular cataract patients and suggested that intake of vitamin D might reduce incidence of posterior subcapsular cataracts [282]. In corroboration, lanosterol, a crucial intermediate in the biosynthesis of steroids and vitamin D, reduced protein aggregation due to mutant crystallin in in vitro tests and cell transfection methods. Moreover, lanosterol attenuated cataract and increased lens transparency in cataractous lenses from rabbits and dogs, thus diminishing severity of cataract, in vivo [283]. Taken together, these studies suggest a significant role for vitamin D in lens transparency.

## 5. Conclusions

Although cataract has been the leading cause of preventable blindness worldwide for many decades, pharmacological strategies to mitigate, prevent, or cure this blinding disease have remained elusive. With the projected increase in life expectancies, the number of people affected with cataract is predicted to increase worldwide. The prognosis of the disease is at best oblique in low income nations that lack easy and affordable access to cataract surgery. Therefore, the potential therapeutic and economic benefits of pharmacological cataract treatments are immeasurable, ranging from a reduction in economic burden to better quality of life. This review presents a wide range of compounds, including antioxidants and herbal remedies/extracts that have exhibited anti-cataract activity in vitro, ex vivo, and in vivo. So far, compounds that possess antioxidant and free radical scavenging activity have shown tremendous potential in these experimental studies. The ready accessibility of plant-derived compounds/mixtures renders them an attractive treatment choice, especially in developing nations where cost is likely to hinder the development and patient use of medication. However, none of these drug candidates have translated into US FDA-approved anti-cataract eye drops or remedies that can prevent, deter, or cure cataracts in humans. Therefore, further research is necessary to establish efficacy and safety profile of these herbal remedies and antioxidants, standardize drug formulations, and conduct double-blinded studies to justify clinical application in humans. It is interesting to note that the Age-Related Eye Disease Study 2 (AREDS2) failed to demonstrate a significant deterrent in the progression of age-related cataract using lutein/zeaxanthine supplementation [284]. Similarly, an extensive Cochrane review did not identify a significant anti-cataract effect of vitamin supplementation in humans [285]. It is conceivable that improvement in drug delivery strategies could enhance anti-cataract efficacy in the prevention and/or treatment of cataracts. Additionally, there is a need to delineate the exact mechanisms involved in the process of formation of cataracts in order to identify new therapeutic targets and new drug candidates that can be refined for therapeutic and/or prophylactic use in humans.

## Figures and Tables

**Table 1:** Type of cataract and their respective causes and risks [10].

Type of Cataracts	Causes	Vulnerable Population
**Congenital and developmental**	Heredity, gestational maldevelopment of lens, maternal malnutrition, infection, drugs, radiation, fetal/infantile factors-anoxia, metabolic disorders, birth trauma, malnutrition, congenital anomalies, idiopathic	It may occur since birth or from infancy to adolescence
**Age-related**	Senescent changes, dehydration, systemic diseases, smoking, oxidative stress, and lack of essential dietary elements	Elderly persons, mostly those over the age of 50 years
**Traumatic**	Some physical damage to the eye lens capsule, penetration of foreign object	People working in hazardous conditions such as welders and those in glass furnaces
**Complicated**	Complications of some chronic inflammatory and degenerative eye diseases	Patients of skin diseases, allergy, uveitis, glaucoma, diabetes, emphysema, asthma
**Metabolic**	Metabolic disorders—diabetes mellitus, galactosemia	Persons deficient in certain enzymes and hormones
**Toxic**	Certain toxicants and drugs- Steroids, NSAID’s	People on steroid therapy and toxic drugs
**Radiation and electrical**	Infra-red rays, x-rays, ultra-violet rays, and powerful electric current	Persons who encounter excess sunlight, artificial radiations, high voltage

Used with permission from Gupta VB (2017) [10].

**Table 2:** Crystallins identified in human lens [17].

Protein	Size (Da)	Residues	ΔG (kJ/mol)	Gene	Chromosomal Location
**αA**	19 909	173	27	*CRYAA*	21q22.3
**αB**	20 159	175	21	*CRYAB*	11q23.1
**βA1**	23 191	198	–	*CRYBA1*	17q11.2
**βA2**	21 964	196	–	*CRYBA2*	2q35
**βA3**	25 150	215	58	*CRYBA1*	17q11.2
**βA4**	22 243	195	–	*CRYBA4*	22q12.1
**βB1**	27 892	251	67	*CRYBB1*	22q12.1
**βB2**	23 249	204	49	*CRYBB2*	22q11.23
**βB3**	24 230	211	–	*CRYBB3*	22q11.23
**γC**	20 747	173	36	*CRYGC*	2q33.3
**γD**	20 607	173	69.4	*CRYGD*	2q33.3
**γS**	20 875	177	43.9	*CRYGS*	3q27.3

Daltons (Da); Gibbs free energy (ΔG [kJ/mol]). Copyright Elsevier (used with permission from Elsevier).

**Table 3:** Summary of in vitro and ex vivo studies of cataractogenesis.

Class	Drugs Tested	Cataract Stimuli	Tissue	Pharmacological Action	Ref
**Antioxidants**	GSH	H_2_O_2_ (10 mM)	Goat lenses	Increased lenticular antioxidant defense enzymes and decreased malondialdehyde levels	[87]
	Ascorbic acid and GSH	Incubation in riboflavin and exposure to sunlight	Bovine lens soluble proteins	Reduced structural crosslinking and proteolytic instability of lens crystallins	[88]
	Alpha-tocopherol, lutein and zeaxanthin	H_2_O_2_ (100 µM)	Human lens epithelial cells	Alpha-tocopherol, lutein and zeaxanthin protected lens protein, lipid, and DNA from oxidative damage. Unlike α-tocopherol, lutein and zeaxanthin did not mitigate GSH depletion.	[89]
	Vitamin C or vitamin E	Buthionine sulfoximine (25–200 µM) treatment followed by H_2_O_2_ (0–800 µM)	Rabbit lens epithelial cells	Pretreatment with vitamin C (25–50 µM) or vitamin E (5–40 µM), restored the resistance of GSH-depleted cells to H_2_O_2_ upholding GSH in its reduced form.	[90]
	Alpha- tocopherol	Glucose (55 mM)	Goat lenses	Increased water-soluble protein content and Na^+^-K^+^-ATPase activity while reducing malondialdehyde levels.	[91]
	Aminothiol WR-1065 and anetholedithiolethione (20 µM)	X-ray irradiation (10 Gy at rate of 2 Gy/min)	Bovine lens epithelial cells	Increased GSH levels and cell viability accompanied by decreased HO fluorescence and lower proportion of cells with apoptotic morphology.	[93]
	Alpha lipoic acid	H_2_O_2_ (0.2 mM)	Adult Sprague-Dawley rat lenses	Inhibited lens’ epithelial cell apoptosis and activated lenticular anti-oxidative enzymes.	[99]
**Ketoacids and amino acid antioxidants**	Pyruvate, alpha ketoglutarate, oxaloacetate	H_2_O_2_ (10 mM)	Goat lenses	Pyruvate (10 mM), alpha ketoglutarate (20 mM) and oxaloacetate (20 mM) decreased lenticular malondialdehyde while augmenting GSH-Px activity.	[104]
	Pyruvate	H_2_O_2_ (10 mM)	Goat lenses	Increased lenticular antioxidant defense enzymes and decreased malondialdehyde levels	[87]
	Pyruvate	H_2_O_2_ (2 mM)	Sprague-Dawley rat lenses	Decreased water insoluble proteins (urea soluble) level and prevented loss of gamma crystallin fraction.	[105]
	Ketoacids and amino acids	H_2_O_2_ (1 mM)	Goat lens	All amino acids (1 mM) protected against GSH depletion except l-tyrosine and l-phenylalanine. All amino acids prevented oxidative stress-induced lens protein aggregation except L aspartic acid.	[107]
	*N*-acetylcysteine amide	Exposure to hyperoxia-	Rabbit lenses	Increased GSH and water-soluble protein content. However, it lowered Na^+^, K^+^-ATPase, and CAT activity.	[112]
	*N*-acetylcysteine amide	Dexamethasone (5 µM)	Sprague-Dawley rat lenses	Elevated GSH/GSSG ratio and limiting lipid peroxidation	[113]
	Acetyl-l-carnitine	Sodium Selenite (100 µM)	Wistar rat lenses	Augmented CAT and GSH-Px activity while reducing malondialdehyde levels.	[116]
	Propolis	Glucose (55 mM)	Rat lens epithelial cells	Propolis (5 and 50 μg/mL) attenuated both the glucose (55 mM)-induced elevation in the expression of reactive oxygen species and elevation in cell viability	[117]
**Plant-derived compounds and Herbal remedies**	Quercetin	Glucose (55 mM)	Goat lenses	Increased water-soluble protein content and Na^+^-K^+^-ATPase activity while reducing malondialdehyde levels.	[91]
	Chrysin, a flavonoid present in honey	Sodium selenite (100 µM/mL)	Wistar rat lenses	Chrysin (200 µM/mL) prevented cataractogenesis. Increased activity of calpain and lenticular preferred calpain (Lp82), as well as mRNA transcript levels of genes that encode m-calpain and Lp82. Lowered calcium transporter proteins and lenticular apoptotic-cascade proteins along with mRNA transcripts of the genes.	[120]
	Epigallocatechin-3-gallate (EGCG), a polyphenol derived from green tea	H_2_O_2_	Human γ-crystallin	EGCG attenuated and reversed peroxide-induced aggregation of αA(66–80), a peptide fragment derived from αA-crystallin peptide	[122]
	Green tea (*Camellia sinensis*) leaves extract	Sodium selenite (100 µM)	Wistar rat lenses	Preserved SOD, GSH-Px, and CAT activities	[123]
	Drevogenin D, a triterpenoid aglycone from *Dregeavolubilis*	Sodium selenite (100 µM)	Rat lenses	Increased activity of SOD, CAT, GSH-Px, and GSH-Rx. It augmented the level of reduced GSH and protein sulfhydryl, while it reduced lipid peroxidation.	[127]
	Aqueous extract of *Trigonellafoenum-graecum* (Fenugreek)	Sodium selenite (100 µM)	Wistar rat lenses	Restored GSH and activities of SOD, GSH-Px and GST while decreasing malondialdehyde levels.	[131]
	A herbal preparation—Triphala (composed of *Emblica officinalis, Terminalia chebula*, and *Terminalia belerica*)	Sodium selenite (100 µM)	Wistar rat lenses	Restored GSH content and activities of SOD, CAT, GSH-Px and GST while malondialdehyde levels were decreased.	[132]
	Ethanol extract of Moringa oliefera	Glucose (55 mM)	Goat lenses	Extracts (200 µg/mL and 500 µg/mL) reduced malondialdehyde levels and increased lenticular CAT, GSH, total and soluble protein.	[136]
	Hydro-ethanolic leaf extract of *Abutilon indicum*	Glucose (55 mM)	Goat lenses	Extract (500 µg/mL) reduced malondialdehyde level and increased total protein content and SOD activity.	[140]
	Ethanol extract of *Zingiberofficinale*	Glucose (55 mM)	Goat lenses	Extract (100, 300, and 500 ng/mL) increased protein (total and water-soluble proteins) content and Na^+^-K^+^-ATPase activity while reducing malondialdehyde levels.	[141]
	Aqueous extract of Seabuckthorn (*Hippophaerhamnoides* L.) leaves	H_2_O_2_ (0.5 mM)	Goat lenses	Reinstated the level of SOD and GSH while reducing malondialdehyde levels	[144]
	Aqueous leaf extract of *Abiespindrow*	H_2_O_2_	Goat lenses	Extracts (5, 10, 15, and 20 mg/mL) increased SOD, GSH, total protein content while lowering malondialdehyde levels proportionally with increase in concentration.	[147]
	Fruit extract *of Luffa cylindrica*	H_2_O_2_ (0.5 mM)	Goat lenses	Increased SOD, GSH, and total protein content while lowering malondialdehyde content.	[151]
	*Ocimum sanctum*	Sodium selenite (100 µM)	Wistar rat lenses	Increased SOD, GSH-Px, GST, and CAT.	[153]

**Table 4:** Summary of in vivo studies of cataractogenesis.

Drug		Cataract Stimuli	Animal Model	Mode of Application	Pharmacological Action	Ref
**Antioxidants**	Vitamin C (Ascorbic acid)	Sodium selenite (20 μmol/kg)	White New Zealand rabbits	Subcutaneous injection	Decreased cataractogenesis by 40%	[162]
		Sodium selenite, 100 µL of 20 μmol/kg	Sprague–Dawley rats	Subcutaneous injection	Subcutaneous 0.1 mL of vitamin C (0.3 mM) injection on 8th day postpartum increased concentration of total protein and soluble protein. Comparable electrophoretic pattern of lens proteins to untreated.	[163]
		10% dietary galactose	Guinea pigs	Dietary	Intensified the loss of Na^+^-K^+^ ATPase activity in the lens capsule-epithelium caused by galactose feeding. Oxidized GSH was not detectable in the lens capsule epithelia. Hexose monophosphate shunt activity was not elevated in lenses of pigs during the first hour of culture after euthanasia	[164]
		Sodium selenite (20 μmol/kg)	Sprague–Dawley rats	Dietary	Ascorbic acid attenuated onset of cataract and loss in chaperone activity.	[165]
	Vitamin E	Sodium selenite (20 μmol/kg)	Sprague–Dawley rats	Subcutaneous injection	Vitamin E attenuated selenite-induced onset of cataract and the corresponding loss in chaperone activity.	[165]
		Prednisolone acetate	Brown–Norway rats	Eye drops	Vitamin E attenuated steroid-induced cataract formation probably due to its antioxidant effect and on the stability of the lens fiber membrane.	[167]
		Ultraviolet B (UVB) radiation	Albino Sprague–Dawley rats	Dietary	Vitamin E attenuated intensity UVB-induced opacity and enhanced lenticular GSH content.	[169]
		Streptozotocin (55 mg/kg)	Wistar rats	Dietary	Vitamin E delayed onset of advanced cataracts	[171]
	Vitamin E- and selenium	SDZ ICT 322 (selective 5-HT3 antagonist)	Wistar rats	Dietary	Deficiency of vitamin E and selenium accelerated onset of cataracts and enhanced lipid peroxidation	[168]
	Alpha-lipoic acid	Fructose	Sprague–Dawley albino rats	Gavage	Retarded onset and progression of cataract. Increased CAT, SOD, GSH-Px, GSH and total protein. It also increased activity of Ca^2+^ ATPase activity while it reduced malondialdehyde and Ca^2+^.	[172]
		l-buthionine(S,R)-sulfoximine	Sprague–Dawley rats	Intraperitoneal injection	Increased lenticular GSH, ascorbate, and vitamin E.	[173]
	Stobadine	Streptozotocin (55 mg/kg)	Wistar rats	Dietary	Reduced plasma malondialdehyde and replenished lenticular Sulfhydryl groups.	[171]
	Melatonin (4 mg/Kg)	Buthionine sulfoximine (3 mmol/kg)	New born rats	Intraperitoneal injection	Inhibited cataract formation in rats evidenced with increased total GSH possibly due to its free radical property or stimulated GSH production.	[178]
**Minerals and trace elements**	Zinc sulfate	Sodium selenite	Rabbits	Eye drops	Retard opacities progression and lowered opacity score.	[183]
	Ebselen	Sodium selenite (30 nmol/kg)	Sprague–Dawley rat s	Subcutaneous injection	Increased GSH levels while it lowered malondialdehyde levels and total nitrite level.	[187]
**Ketoacids and amino acids**	Sodium pyruvate	streptozotocin (55 mg/kg)	Sprague–Dawley rats	Dietary	Decreased levels of glycated proteins, sorbitol, malondialdehyde while it increased activity of the cation pump.	[188]
	Pyruvate	Sodium selenite (0.5 µmoles)	Sprague–Dawley rats	Intraperitoneal injection	It prevented cataractogenesis and its level was increased in the aqueous humor.	[105]
	l-cysteine	Sodium selenite, 100 µL of 20 μmol/kg	Sprague–Dawley rats	Subcutaneous injection	Subcutaneous 0.1 mL of l-cysteine (0.05 μmol) on 8th day postpartum increased concentration of total protein and soluble protein. Comparable electrophoretic pattern of lens proteins to untreated.	[158]
	*N*-acetylcysteine	Sodium selenite subcutaneously (30 nmol/g).	Sprague–Dawley rat	Intraperitoneal injection	Reduced cataract formation by 71.4%. Increased lenticular and serum GSH while reducing lenticular protein carbonyl and lenticular and serum malondialdehyde level.	[189]
		Triamcinolone acetonide (1 mg)	Wistar–Albino rats	Intraperitoneal injection	Increased lenticular GSH and GSH-Px while it reduced the level of protein carbonyl and malondialdehyde.	[190]
	*N*-acetylcysteine amide and GSH ethyl ester	Streptozotocin (65 mg/kg)	Sprague–Dawley rats	Eye drops	Inhibited cataract progression at an early after which activity declined. Did not increase GSH-Px and CAT. Increased glycation levels and thiols.	[191]
	*N*-acetylcysteine amide	l-buthionine-(S,R)-sulfoximine	Wistar rats	Intraperitoneal injection	Replenished GSH levels of replenished and limited protein carbonylation, lipid peroxidation, and redox system components.	[193]
		Sodium selenite	Wistar rats	Intraperitoneal injection	Reversed cataract grade. Increased GSH level, thioltransferase activity, m-calpain activity, and m-calpain levels while it reduced malondialdehyde level, GSH-Px enzyme activity, and calcium levels	[192]
		Sodium selenite	Wistar rats	Eye drops	Reversed cataract grade. Increased GSH level, thioltransferase activity, m-calpain activity, and m-calpain levels while it reduced malondialdehyde level, GSH-Px enzyme activity, and calcium levels	[192]
	Acetyl-l-carnitine	Sodium selenite	Wistar rats	Intraperitoneal injection	Increased GSH content as well as GST and GSH-Px activity while it lowered malondialdehyde level. It also increased staining intensity of isozyme bands for SOD and GSH-Px.	[194]
**Plant-derived compounds and herbal remedies**	water-insoluble antioxidants (lutein, zeaxanthin hesperetin, quercetin, anthocyanin, β-carotene, and α-tocopherol) and water-soluble antioxidants (ascorbic acid, cyanidin)	Sodium selenite (20 μmol/Kg)	Sprague Dawley rats	Subcutaneous injection	Maintained activity of chaperone activity in water soluble lens proteins.	[165]
	Rutin	Sodium selenite (19 µmol/kg)	Wistar rats	Intraperitoneal injection	Inhibited lipid peroxidation and increased activity of SOD, CAT, GSH-Px, and, GST.	[197]
	Hesperetin (flavonoid)	Sodium selenite (20 μmol/Kg)	Sprague–Dawley rats	Subcutaneous injection	Increased expression of the of filensin (94 and 50 kDa forms). Interestingly, these forms of filensin Increased lenticular GSH and ascorbic acid levels.	[200]
	Hesperetin (flavonoid) and derivatives	Sodium selenite (20 μmol/Kg)	Sprague–Dawley rats	Subcutaneous injection	Mitigated decreased lens chaperone activity and α-crystallin water solubility.	[201,202]
	Ellagic acid	Sodium selenite (19 µmol/kg)	Wistar rats	Intraperitoneal injection	Lenticular and erythrocytic GSH were increased while it reduced lenticular malondialdehyde and calcium content.	[206]
	Green tea (Camellia sinensis)	Sodium selenite (0.25 µmol/Kg)	Wistar rats	Intraperitoneal injection	Decreased cataractogenesis by 66.67%	[207]
	*Green or black tea extracts*	Sodium selenite (2.2 mg/kg)	Wistar rats	Subcutaneous injection	Scavenged reactive oxygen species and prevented oxidative cross-linking of proteins and single strand breakage of DNA.	[207]
		Streptozotocin (65 mg/Kg)	Sprague–Dawley rats	Drinking water	Hypoglycemic effect retreaded cataract formation.	[208]
	Caffeine	Ultra-violate-B radiation	Sprague–Dawley rats	Eye drops	Reduced caspase-3 and lens sensitivity to ultra-violate-B by 1.23 times.	[211]
		Sodium selenite (15 µmol/kg)	Sprague–Dawley rats	Gastric intubation	Reduced lenticular level of malondialdehyde, total nitric oxide, Ca+-ATPase, tumor necrosis factor-α, interleukin-1β, SOD, while it increased lenticular total protein, reduced GSH, and CAT.	[212]
	Caffeine and pyrocatechol	Sodium selenite (20 μmol/Kg)	Sprague–Dawley rats	Subcutaneous injection	Maintained activity of chaperone activity in water soluble lens proteins.	[165]
	β-carotene	Sodium selenite (20 μmol/Kg)	Sprague–Dawley rats	Subcutaneous injection	Maintained activity of chaperone activity in water soluble lens proteins.	[165]
	Lycopene	Dietary 30% galactose	Wistar rats	Intraperitoneal injection	Reduced selenite induced cataract by 89% and reduced onset and progression of galactose induced cataract was observed with oral feeding of lycopene. Only 35% of the eyes developed mature cataract as opposed to 100% in the control group	[217]
		Dietary 30% galactose	Wistar rats	Intraperitoneal injection	Reduced selenite induced cataract by 89% and reduced onset and progression of galactose induced cataract were observed with oral feeding of lycopene. Only 35% of the eyes developed mature cataract as opposed to 100% in the control group	[217]
	Curcumin	Galactose (30%)	Sprague–Dawley rats	Dietary	Augmented GSH while it reduced malondialdehyde levels. It also inhibited advanced glycation end product formation and protein aggregation.	[218]
		Sodium selenite (30 µM/Kg)	Wistar rats	Subcutaneous injection	Increased activity of SOD and CAT while it reduced malondialdehyde levels and xanthine oxidase activity.	[219]
		Sodium selenite (15 µM/Kg)	Wistar rats	Intraperitoneal injection	Reduced malondialdehyde levels while it increased SOD, GSH-Px, GST, and CAT activity.	[220]
		Streptozotocin (35 mg/Kg)	WNIN rats	Dietary	Reduced malondialdehyde levels, increased reduced GSH, protein carbonyl content and activities of peroxide dismutase, GSH-Px, and glucose-6-phosphate dehydrogenase	[221]
	Turmeric	Streptozotocin (35 mg/kg)	Wistar–NIN rats	Dietary	Reduced lipid peroxidation and protein carbonyl content while it increased GSH and antioxidant enzyme activities.	[221]
	Resveratrol	Sodium selenite (30 nmol/g)	Spraque–Dawley rats	Subcutaneous injection	Increased lenticular and erythrocytic GSH and lowered malondialdehyde levels.	[225]
	*Heliotropiumindicum* extract	Sodium selenite (15 µmol/kg)	Sprague–Dawley rats	Oral	Preserved epithelial and lens fiber integrity, aquaporin 0, alpha A and B crystallins, total lens proteins, and lenticular GSH levels. It also showed free radicals scavenging activity and inhibited lipid peroxidation.	[228]
	Hydroalchoholic extract of *Echium amoenum*	Sodium selenite (30 nmol/kg)	White rats	Intraperitoneal injection	Improved cataract grade and optical clarity of lenses.	[230]
	H636 grape seed proanthocyanidin extract	Sodium selenite (30 nmol/g)	Spraque–Dawley rats	Oral	Increased lenticular GSH content and reduced malondialdehyde.	[233]
	*Cassia tora* Linn. leaves	Sodium selenite (4 μg/g)	Spraque–Dawley rats	Oral	Reduced oxidative stress index, prevented structural crystallin loss, and increased total peroxide level.	
	*Vaccinium corymbosum* leaf decoction (chlorogenic acid, quercetin, rutin, isoquercetin and hyperoside)	Sodium selenite (20 µmole/Kg)	White rats	Intraperitoneal injection	Prevented oxidative attack and calpain activation, protein loss and aggregation.	[236]
	*Emilia sonchifolia* (flavonoid fraction)	Sodium selenite (4 mg/kg)	Rat	Intraperitoneal injection	Increased GSH, activities of SOD and CAT while thiobarbituric acid reacting substances were reduced.	
	*Brassica oleracea var. italica* (flavonoid fraction)	Sodium selenite (4 mg/kg)	Sprague–Dawley rats	Intraperitoneal injection	Increase SOD, CAT, GSH, Ca^2+^ ATPase while it reduced, calcium, calpains and lipid peroxidation product-thiobarbituric acid reacting substances	[241]
	Lupeol, a flavonoid from the plant, *Vernonia cinereal* plant	Sodium selenite (25 μg/g)	Sprague–Dawley rats	Oral	Reduced lipid peroxidation and protein oxidation. Upheld activity of. Lenticular SOD, CAT, GSH-Px, GSH-Rx, GST, and GSH content.	[243]
	Methanolic extract of *Allium sativum*	Streptozotocin (34 mg/kg body weight)	Wistar rats	Forced gut- feeding	Delay in onset of cataracts; Restoration of lenticular GSH, GSH-Px and SOD activities	[245]
	Aqueous extract of *Allium cepa*	Sodium selenite (30 nmol/g body weight)	Wistar albino rats	Eye drops	The allium aqueous extract of garlic preserved optical clarity; Allium-treated lenses exhibited higher total antioxidants and higher GSH-Px and SOD activities	[246]
	*Pinus densiflora* bark extract	Sodium selenite (18 μmol/kg)	Sprague–Dawley rats	Gastric intubation	Increased water-soluble protein and GSH content, SOD, GSH-Px, and CAT activity while it lowered water-insoluble protein, malondialdehyde, and Ca^2+^-ATPase. It inhibited *m*-calpain-induced proteolysis and apoptosis.	[249]
	*Ocimum sanctum*	Sodium selenite (25 µmole/kg)	Wistar rats	Intraperitoneal injection	Prevented lens protein insolubilization.	[153]
	*Crocus sativus* stigmas (saffron) extra	Sodium selenite (20 µmol/kg)	Wistar rats	Intraperitoneal injection	Increased activities of SOD, GSH-Px, CAT and GSH content. Halted lipid peroxidation, protein oxidation, and proteolysis and insolubilization of water-soluble proteins.	[251]
	Propolis	d-galactose (15% or 25%)	Sprague–Dawley albino rats	Oral (dietary)	Reduced reactive oxygen species and improved epithelial cell viability.	[117]
	An aqueous extract of *Pterocarpus marsupium Linn bark* and alcoholic extract of *Trigonellafoenum-graecum Linn* seeds	Alloxan 120 mg/Kg	Rats	Gastric tube	Showed antihyperglycemic effect and reduced opacity index.	[253]
	*Ginkgo biloba*	Total-cranium irradiation (5 Gy)	Sprague–Dawley rats	Oral	Increase the activities of SOD and GSH-Px while it reduced and significantly decreased malonaldehyde content.	[256]
	A herbal preparation-Triphala (*Emblica officinalis, Terminalia chebula*, and *Terminalia belerica*)	Sodium selenite (100 µM)	Wistar rat lenses	Intraperitoneal injection	Delayed onset and progression of cataracts	[132]

## Abbreviations

ACE	Angiotensin converting enzyme
ATPase	Adenosine triphosphatase
BSO	Buthionine sulfoximine
CAT	Catalase
CNS	Central nervous system
Cu	Copper
EC	Extracellular
EGCG	Epigallocatechin-3-gallate
GSH	Glutathione
GSH-Px	Glutathione peroxidase
GSPE	Grape seed proanthocyanidin extract
GSH-Rx	Glutathione reductase
GSSG	Glutathione disulfide
GST	Glutathione-S-Transferase
K^+^	Potassium cation
Mn-SOD	Mitochondrial-superoxide dismutase
Na^+^	Sodium cation
Na^+^-K^+^-ATPase	sodium-potassium adenosine triphosphatase
NADPH	Nicotinamide adenine dinucleotide phosphate
NSAIDs	Non-steroidal anti-inflammatory drugs
SOD	Superoxide dismutase
TGF-β	Transforming growth factor beta
UV	Ultraviolet
UVA	Ultraviolet A
UVB	Ultraviolet B
Zn	Zinc
