# Correction: Heruye et al. Current Trends in the Pharmacotherapy of Cataracts. *Pharmaceuticals* 2020, *13*, 15

**DOI:** 10.3390/ph17070947

**Published:** 2024-07-15

**Authors:** Segewkal H. Heruye, Leonce N. Maffofou Nkenyi, Neetu U. Singh, Dariush Yalzadeh, Kalu K. Ngele, Ya-Fatou Njie-Mbye, Sunny E. Ohia, Catherine A. Opere

**Affiliations:** 1 Department of Pharmacology & Neuroscience, School of Medicine, Creighton University, 2500 California Plaza, Omaha, NE 68178, USA; 2 Department of Pharmacy Sciences, School of Pharmacy and Health Professions, Creighton University, 2500 California Plaza, Omaha, NE 68178, USA; 3 College of Southern Nevada, Las Vegas, NV 89146, USA; 4 Department of Biology/Microbiology/Biotechnology, Federal University Ndufu Alike Ikwo, Abakaliki, Nigeria; 5 Department of Pharmaceutical Sciences, College of Pharmacy and Health Sciences, Texas Southern University, Houston, TX 77004, USA

In the original publication [1], (1) reference 241. Lija, Y.; Biju, P.G.; Reeni, A.; Cibin, T.R.; Sahasranamam, V.; Abraham, A. Modulation of selenite cataract by the flavonoid fraction of Emilia sonchifolia in experimental animal models. Phytother. Res. 2006, 20, 1091–1095; (2) reference 236. Sreelakshmi, V.; Abraham, A. Cassia tora leaves modulates selenite cataract by enhancing antioxidant status and preventing cytoskeletal protein loss in lenses of Sprague Dawley rat pups. J. Ethnopharmacol. 2016, 178, 137–143.; and (3) reference 235. Sreelakshmi, V.; Abraham, A. Anthraquinones and flavonoids of Cassia tora leaves ameliorate sodium selenite induced cataractogenesis in neonatal rats. Food Funct. 2016, 7, 1087–1095. were cited wrongly. The citation should be removed from the manuscript. With this correction, the order of some references has been adjusted accordingly. The authors state that the scientific conclusions are unaffected. This correction was approved by the Academic Editor. The original publication has also been updated.
